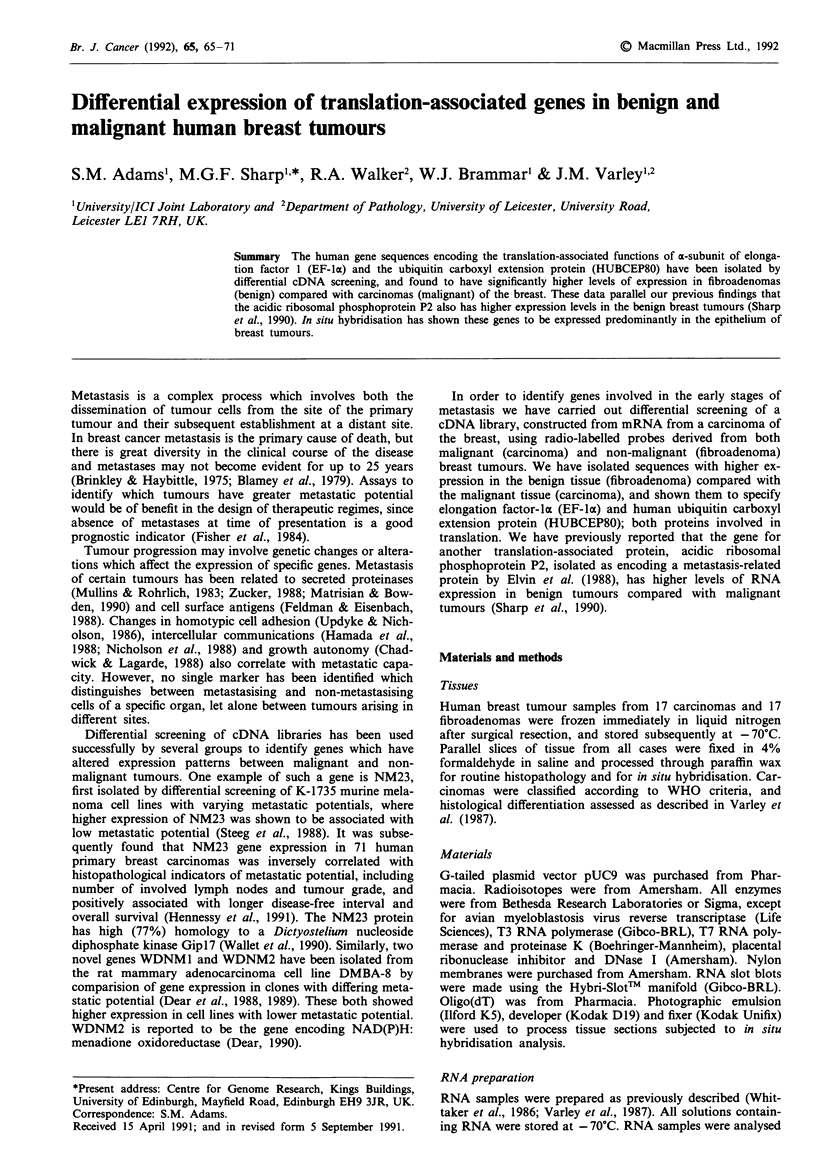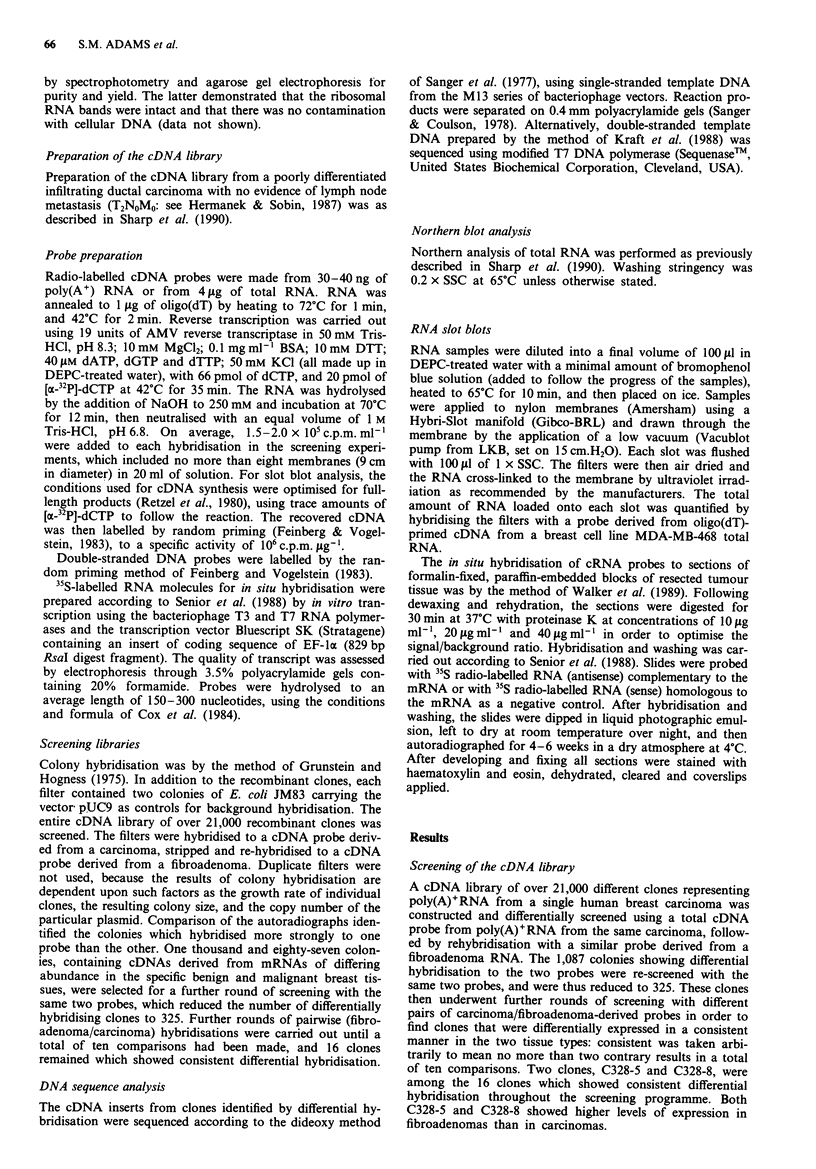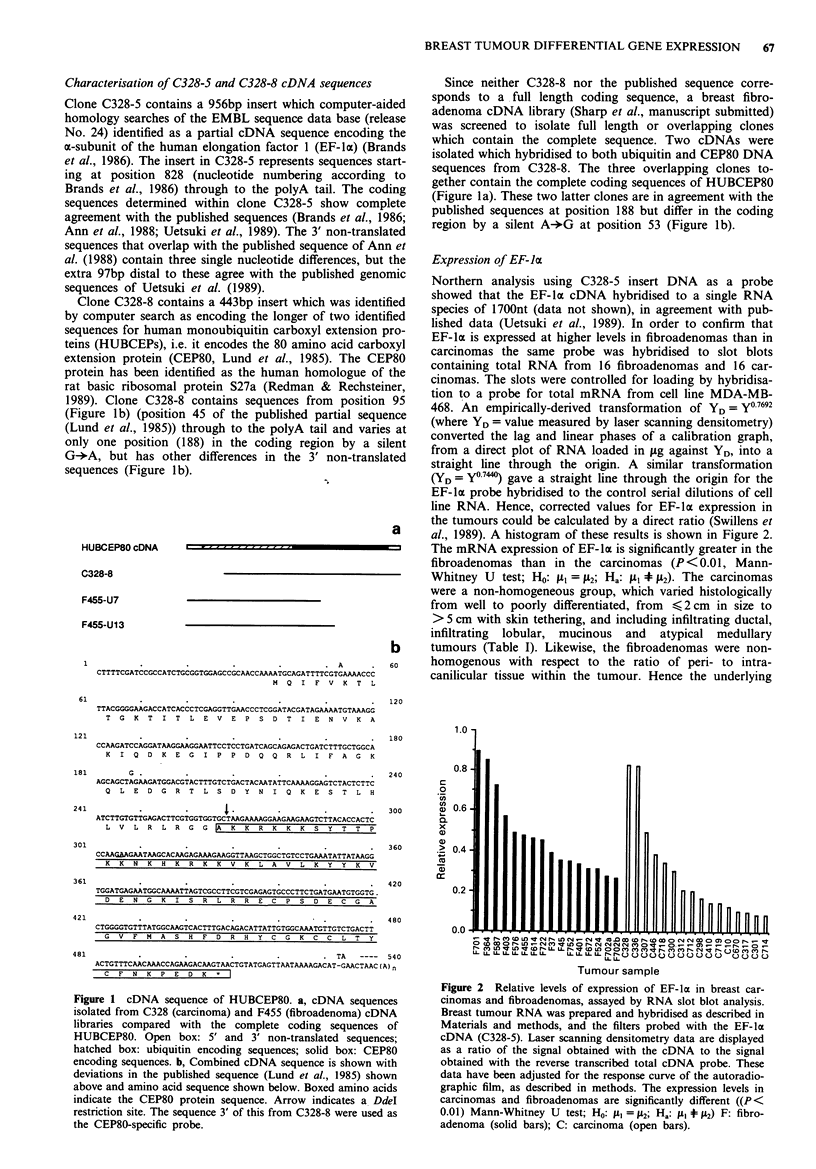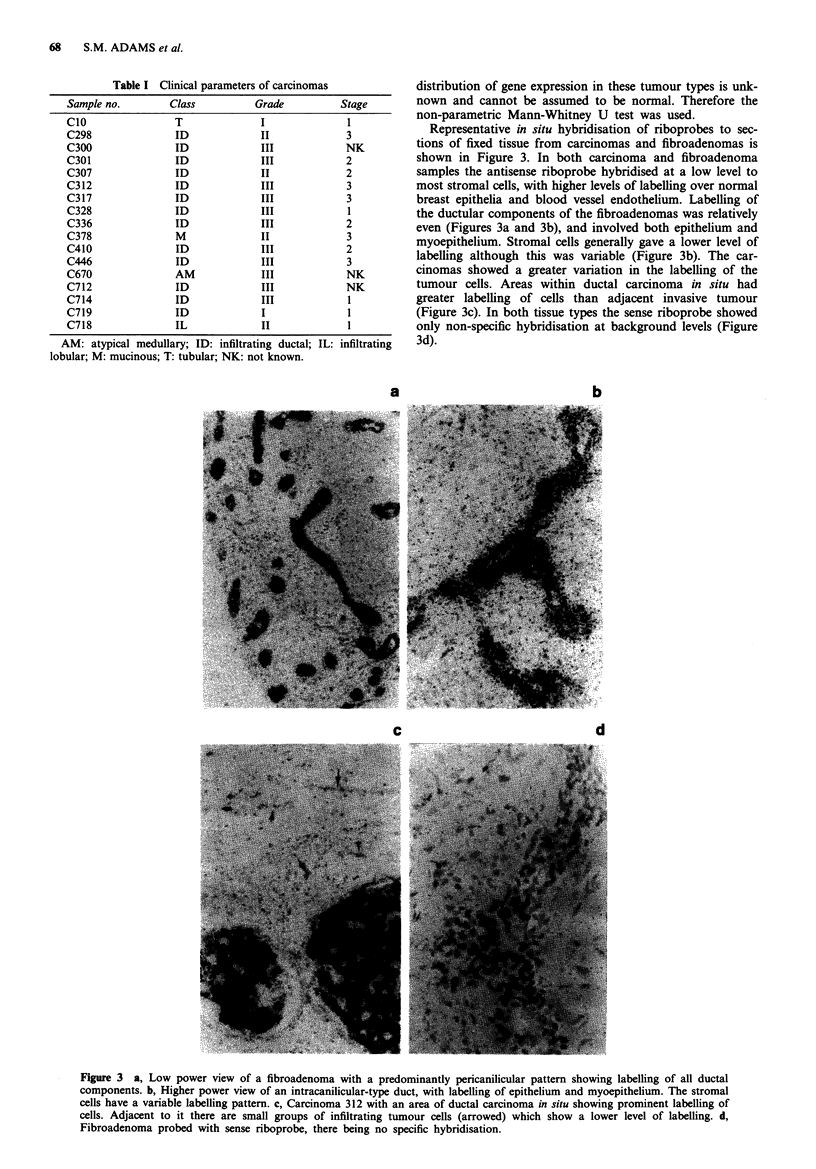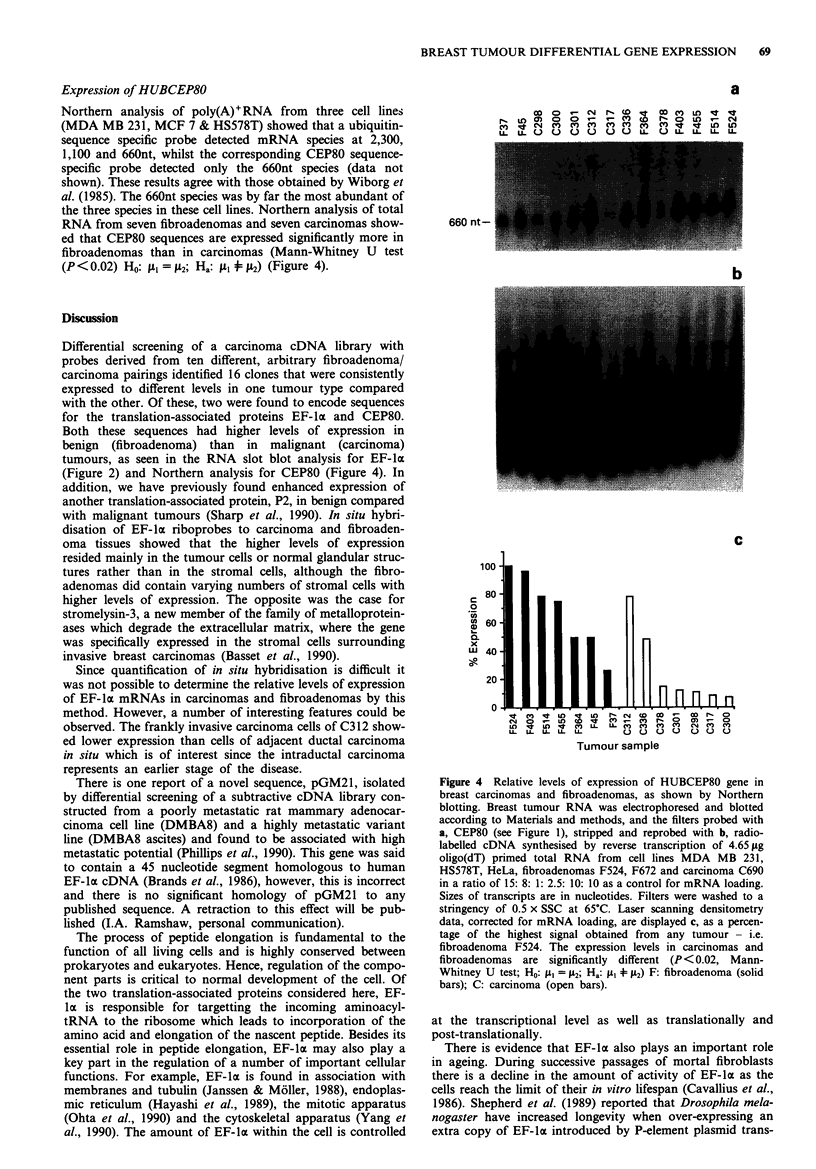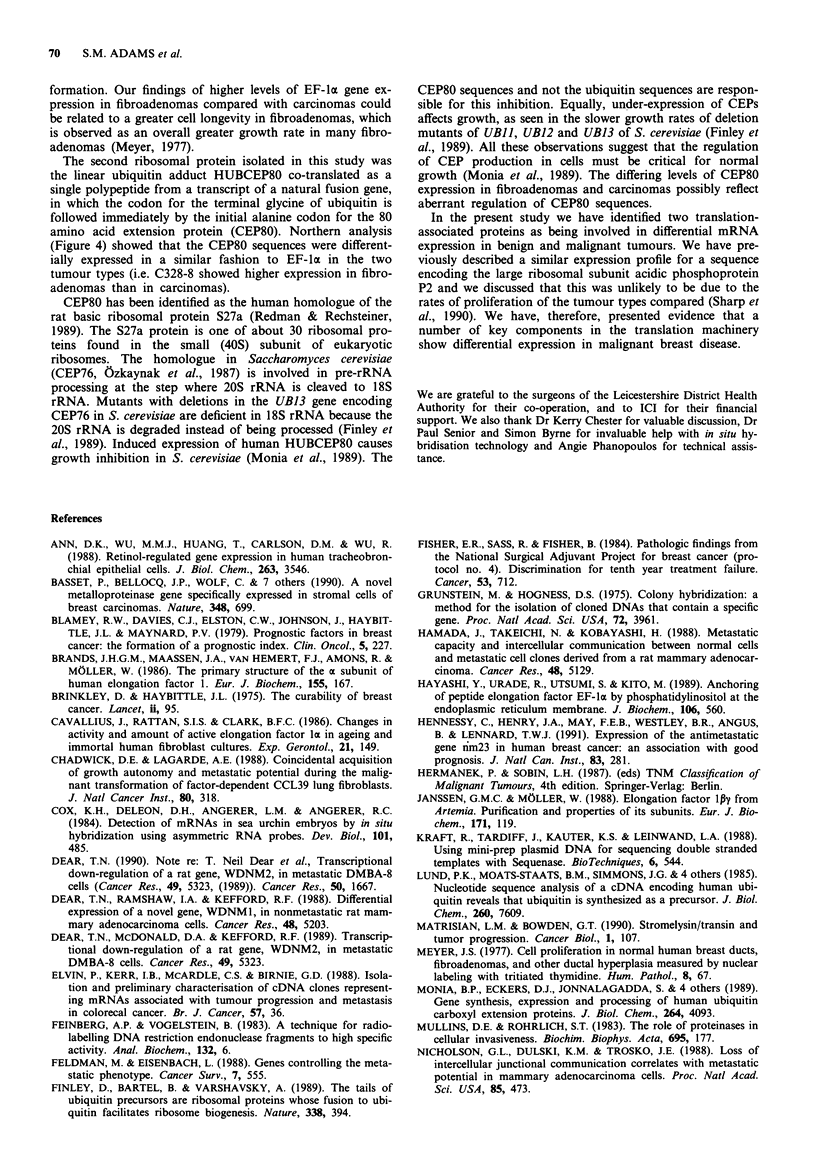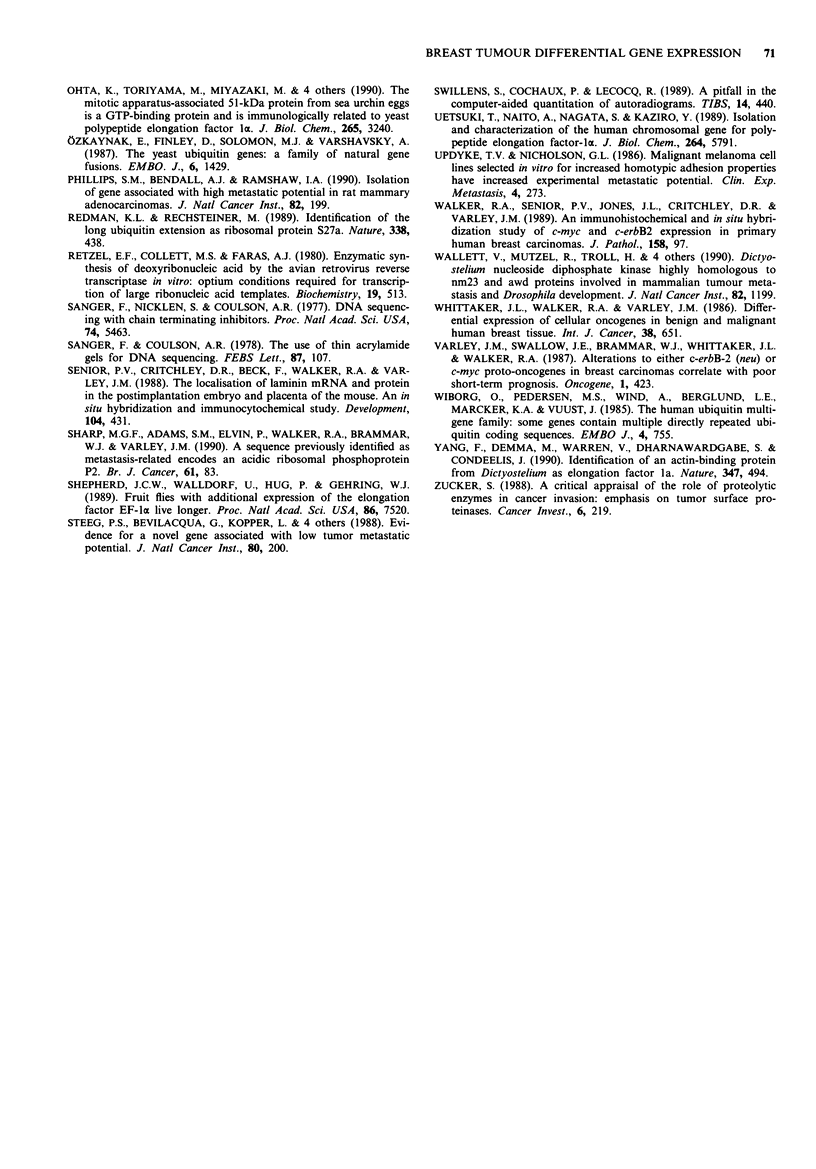# Differential expression of translation-associated genes in benign and malignant human breast tumours.

**DOI:** 10.1038/bjc.1992.12

**Published:** 1992-01

**Authors:** S. M. Adams, M. G. Sharp, R. A. Walker, W. J. Brammar, J. M. Varley

**Affiliations:** University/ICI Joint Laboratory, University of Leicester, UK.

## Abstract

**Images:**


Br. J. Cancer (1992), 65, 65–71 © Macmillan Press Ltd., 1992

### Differential expression of translation-associated genes in benign and malignant human breast tumours

S.M. Adams[1], M.G.F. Sharp[1,*], R.A. Walker[2], W.J. Brammar[1] & J.M. Varley[1,2]

[1]University/ICI Joint Laboratory and [2]Department of Pathology, University of Leicester, University Road, Leicester LE1 7RH, UK.

**Summary** The human gene sequences encoding the translation-associated functions of α-subunit of elongation factor 1 (EF-1α) and the ubiquitin carboxyl extension protein (HUBCEP80) have been isolated by differential cDNA screening, and found to have significantly higher levels of expression in fibroadenomas (benign) compared with carcinomas (malignant) of the breast. These data parallel our previous findings that the acidic ribosomal phosphoprotein P2 also has higher expression levels in the benign breast tumours (Sharp *et al.*, 1990). *In situ* hybridisation has shown these genes to be expressed predominantly in the epithelium of breast tumours.

Metastasis is a complex process which involves both the dissemination of tumour cells from the site of the primary tumour and their subsequent establishment at a distant site. In breast cancer metastasis is the primary cause of death, but there is great diversity in the clinical course of the disease and metastases may not become evident for up to 25 years (Brinkley & Haybittle, 1975; Blamey *et al.*, 1979). Assays to identify which tumours have greater metastatic potential would be of benefit in the design of therapeutic regimes, since absence of metastases at time of presentation is a good prognostic indicator (Fisher *et al.*, 1984).

Tumour progression may involve genetic changes or alterations which affect the expression of specific genes. Metastasis of certain tumours has been related to secreted proteinases (Mullins & Rohrlich, 1983; Zucker, 1988; Matrisian & Bowden, 1990) and cell surface antigens (Feldman & Eisenbach, 1988). Changes in homotypic cell adhesion (Updyke & Nicholson, 1986), intercellular communications (Hamada *et al.*, 1988; Nicholson *et al.*, 1988) and growth autonomy (Chadwick & Lagarde, 1988) also correlate with metastatic capacity. However, no single marker has been identified which distinguishes between metastasising and non-metastasising cells of a specific organ, let alone between tumours arising in different sites.

Differential screening of cDNA libraries has been used successfully by several groups to identify genes which have altered expression patterns between malignant and non-malignant tumours. One example of such a gene is NM23, first isolated by differential screening of K-1735 murine melanoma cell lines with varying metastatic potentials, where higher expression of NM23 was shown to be associated with low metastatic potential (Steeg *et al.*, 1988). It was subsequently found that NM23 gene expression in 71 human primary breast carcinomas was inversely correlated with histopathological indicators of metastatic potential, including number of involved lymph nodes and tumour grade, and positively associated with longer disease-free interval and overall survival (Hennessy *et al.*, 1991). The NM23 protein has high (77%) homology to a *Dictyostelium* nucleoside diphosphate kinase Gip17 (Wallet *et al.*, 1990). Similarly, two novel genes WDNM1 and WDNM2 have been isolated from the rat mammary adenocarcinoma cell line DMBA-8 by comparision of gene expression in clones with differing metastatic potential (Dear *et al.*, 1988, 1989). These both showed higher expression in cell lines with lower metastatic potential. WDNM2 is reported to be the gene encoding NAD(P)H: menadione oxidoreductase (Dear, 1990).

*Present address: Centre for Genome Research, Kings Buildings, University of Edinburgh, Mayfield Road, Edinburgh EH9 3JR, UK. Correspondence: S.M. Adams.
Received 15 April 1991; and in revised form 5 September 1991.

In order to identify genes involved in the early stages of metastasis we have carried out differential screening of a cDNA library, constructed from mRNA from a carcinoma of the breast, using radio-labelled probes derived from both malignant (carcinoma) and non-malignant (fibroadenoma) breast tumours. We have isolated sequences with higher expression in the benign tissue (fibroadenoma) compared with the malignant tissue (carcinoma), and shown them to specify elongation factor-1α (EF-1α) and human ubiquitin carboxyl extension protein (HUBCEP80); both proteins involved in translation. We have previously reported that the gene for another translation-associated protein, acidic ribosomal phosphoprotein P2, isolated as encoding a metastasis-related protein by Elvin *et al.* (1988), has higher levels of RNA expression in benign tumours compared with malignant tumours (Sharp *et al.*, 1990).

### Materials and methods

### Tissues

Human breast tumour samples from 17 carcinomas and 17 fibroadenomas were frozen immediately in liquid nitrogen after surgical resection, and stored subsequently at −70°C. Parallel slices of tissue from all cases were fixed in 4% formaldehyde in saline and processed through paraffin wax for routine histopathology and for *in situ* hybridisation. Carcinomas were classified according to WHO criteria, and histological differentiation assessed as described in Varley *et al.* (1987).

### Materials

G-tailed plasmid vector pUC9 was purchased from Pharmacia. Radioisotopes were from Amersham. All enzymes were from Bethesda Research Laboratories or Sigma, except for avian myeloblastosis virus reverse transcriptase (Life Sciences), T3 RNA polymerase (Gibco-BRL), T7 RNA polymerase and proteinase K (Boehringer-Mannheim), placental ribonuclease inhibitor and DNase I (Amersham). Nylon membranes were purchased from Amersham. RNA slot blots were made using the Hybri-Slot™ manifold (Gibco-BRL). Oligo(dT) was from Pharmacia. Photographic emulsion (Ilford K5), developer (Kodak D19) and fixer (Kodak Unifix) were used to process tissue sections subjected to *in situ* hybridisation analysis.

### RNA preparation

RNA samples were prepared as previously described (Whittaker *et al.*, 1986; Varley *et al.*, 1987). All solutions containing RNA were stored at −70°C. RNA samples were analysed by spectrophotometry and agarose gel electrophoresis for purity and yield. The latter demonstrated that the ribosomal RNA bands were intact and that there was no contamination with cellular DNA (data not shown).

*Preparation of the cDNA library*

Preparation of the cDNA library from a poorly differentiated infiltrating ductal carcinoma with no evidence of lymph node metastasis ($T_2N_0M_0$: see Hermanek & Sobin, 1987) was as described in Sharp *et al.* (1990).

*Probe preparation*

Radio-labelled cDNA probes were made from 30–40 ng of poly($A^+$) RNA or from 4 μg of total RNA. RNA was annealed to 1 μg of oligo(dT) by heating to 72°C for 1 min, and 42°C for 2 min. Reverse transcription was carried out using 19 units of AMV reverse transcriptase in 50 mM Tris-HCl, pH 8.3; 10 mM $MgCl_2$; 0.1 mg $ml^{-1}$ BSA; 10 mM DTT; 40 μM dATP, dGTP and dTTP; 50 mM KCl (all made up in DEPC-treated water), with 66 pmol of dCTP, and 20 pmol of [α-$^{32}$P]-dCTP at 42°C for 35 min. The RNA was hydrolysed by the addition of NaOH to 250 mM and incubation at 70°C for 12 min, then neutralised with an equal volume of 1 M Tris-HCl, pH 6.8. On average, $1.5–2.0 \times 10^5$ c.p.m. $ml^{-1}$ were added to each hybridisation in the screening experiments, which included no more than eight membranes (9 cm in diameter) in 20 ml of solution. For slot blot analysis, the conditions used for cDNA synthesis were optimised for full-length products (Retzel *et al.*, 1980), using trace amounts of [α-$^{32}$P]-dCTP to follow the reaction. The recovered cDNA was then labelled by random priming (Feinberg & Vogelstein, 1983), to a specific activity of $10^6$ c.p.m. $\mu g^{-1}$.

Double-stranded DNA probes were labelled by the random priming method of Feinberg and Vogelstein (1983).

$^{35}$S-labelled RNA molecules for *in situ* hybridisation were prepared according to Senior *et al.* (1988) by *in vitro* transcription using the bacteriophage T3 and T7 RNA polymerases and the transcription vector Bluescript SK (Stratagene) containing an insert of coding sequence of EF-1α (829 bp *RsaI* digest fragment). The quality of transcript was assessed by electrophoresis through 3.5% polyacrylamide gels containing 20% formamide. Probes were hydrolysed to an average length of 150–300 nucleotides, using the conditions and formula of Cox *et al.* (1984).

*Screening libraries*

Colony hybridisation was by the method of Grunstein and Hogness (1975). In addition to the recombinant clones, each filter contained two colonies of *E. coli* JM83 carrying the vector pUC9 as controls for background hybridisation. The entire cDNA library of over 21,000 recombinant clones was screened. The filters were hybridised to a cDNA probe derived from a carcinoma, stripped and re-hybridised to a cDNA probe derived from a fibroadenoma. Duplicate filters were not used, because the results of colony hybridisation are dependent upon such factors as the growth rate of individual clones, the resulting colony size, and the copy number of the particular plasmid. Comparison of the autoradiographs identified the colonies which hybridised more strongly to one probe than the other. One thousand and eighty-seven colonies, containing cDNAs derived from mRNAs of differing abundance in the specific benign and malignant breast tissues, were selected for a further round of screening with the same two probes, which reduced the number of differentially hybridising clones to 325. Further rounds of pairwise (fibroadenoma/carcinoma) hybridisations were carried out until a total of ten comparisons had been made, and 16 clones remained which showed consistent differential hybridisation.

*DNA sequence analysis*

The cDNA inserts from clones identified by differential hybridisation were sequenced according to the dideoxy method of Sanger *et al.* (1977), using single-stranded template DNA from the M13 series of bacteriophage vectors. Reaction products were separated on 0.4 mm polyacrylamide gels (Sanger & Coulson, 1978). Alternatively, double-stranded template DNA prepared by the method of Kraft *et al.* (1988) was sequenced using modified T7 DNA polymerase (Sequenase™, United States Biochemical Corporation, Cleveland, USA).

*Northern blot analysis*

Northern analysis of total RNA was performed as previously described in Sharp *et al.* (1990). Washing stringency was 0.2 × SSC at 65°C unless otherwise stated.

*RNA slot blots*

RNA samples were diluted into a final volume of 100 μl in DEPC-treated water with a minimal amount of bromophenol blue solution (added to follow the progress of the samples), heated to 65°C for 10 min, and then placed on ice. Samples were applied to nylon membranes (Amersham) using a Hybri-Slot manifold (Gibco-BRL) and drawn through the membrane by the application of a low vacuum (Vacublot pump from LKB, set on 15 cm.$H_2O$). Each slot was flushed with 100 μl of 1 × SSC. The filters were then air dried and the RNA cross-linked to the membrane by ultraviolet irradiation as recommended by the manufacturers. The total amount of RNA loaded onto each slot was quantified by hybridising the filters with a probe derived from oligo(dT)-primed cDNA from a breast cell line MDA-MB-468 total RNA.

The *in situ* hybridisation of cRNA probes to sections of formalin-fixed, paraffin-embedded blocks of resected tumour tissue was by the method of Walker *et al.* (1989). Following dewaxing and rehydration, the sections were digested for 30 min at 37°C with proteinase K at concentrations of 10 μg $ml^{-1}$, 20 μg $ml^{-1}$ and 40 μg $ml^{-1}$ in order to optimise the signal/background ratio. Hybridisation and washing was carried out according to Senior *et al.* (1988). Slides were probed with $^{35}$S radio-labelled RNA (antisense) complementary to the mRNA or with $^{35}$S radio-labelled RNA (sense) homologous to the mRNA as a negative control. After hybridisation and washing, the slides were dipped in liquid photographic emulsion, left to dry at room temperature over night, and then autoradiographed for 4–6 weeks in a dry atmosphere at 4°C. After developing and fixing all sections were stained with haematoxylin and eosin, dehydrated, cleared and coverslips applied.

### Results

*Screening of the cDNA library*

A cDNA library of over 21,000 different clones representing poly(A)$^+$RNA from a single human breast carcinoma was constructed and differentially screened using a total cDNA probe from poly(A)$^+$RNA from the same carcinoma, followed by rehybridisation with a similar probe derived from a fibroadenoma RNA. The 1,087 colonies showing differential hybridisation to the two probes were re-screened with the same two probes, and were thus reduced to 325. These clones then underwent further rounds of screening with different pairs of carcinoma/fibroadenoma-derived probes in order to find clones that were differentially expressed in a consistent manner in the two tissue types: consistent was taken arbitrarily to mean no more than two contrary results in a total of ten comparisons. Two clones, C328-5 and C328-8, were among the 16 clones which showed consistent differential hybridisation throughout the screening programme. Both C328-5 and C328-8 showed higher levels of expression in fibroadenomas than in carcinomas.

*Characterisation of C328-5 and C328-8 cDNA sequences*

Clone C328-5 contains a 956bp insert which computer-aided homology searches of the EMBL sequence data base (release No. 24) identified as a partial cDNA sequence encoding the α-subunit of the human elongation factor 1 (EF-1α) (Brands *et al.*, 1986). The insert in C328-5 represents sequences starting at position 828 (nucleotide numbering according to Brands *et al.*, 1986) through to the polyA tail. The coding sequences determined within clone C328-5 show complete agreement with the published sequences (Brands *et al.*, 1986; Ann *et al.*, 1988; Uetsuki *et al.*, 1989). The 3′ non-translated sequences that overlap with the published sequence of Ann *et al.* (1988) contain three single nucleotide differences, but the extra 97bp distal to these agree with the published genomic sequences of Uetsuki *et al.* (1989).

Clone C328-8 contains a 443bp insert which was identified by computer search as encoding the longer of two identified sequences for human monoubiquitin carboxyl extension proteins (HUBCEPs), i.e. it encodes the 80 amino acid carboxyl extension protein (CEP80, Lund *et al.*, 1985). The CEP80 protein has been identified as the human homologue of the rat basic ribosomal protein S27a (Redman & Rechsteiner, 1989). Clone C328-8 contains sequences from position 95 (Figure 1b) (position 45 of the published partial sequence (Lund *et al.*, 1985)) through to the polyA tail and varies at only one position (188) in the coding region by a silent G→A, but has other differences in the 3′ non-translated sequences (Figure 1b).

**b**

```
1                                                      .  A     .    60
    CTTTTCGATCCGCCATCTGCGGTGGAGCCGCAACCAAAATGCAGATTTTCGTGAAAACCC
                                            M  Q  I  F  V  K  T  L

61                                                              .   120
    TTACGGGGAAGACCATCACCCTCGAGGTTGAACCCTCGGATACGATAGAAAATGTAAAGG
      T  G  K  T  I  T  L  E  V  E  P  S  D  T  I  E  N  V  K  A

121                                                             .   180
    CCAAGATCCAGGATAAGGAAGGAATTCCTCCTGATCAGCAGAGACTGATCTTTGCTGGCA
      K  I  Q  D  K  E  G  I  P  P  D  Q  Q  R  L  I  F  A  G  K

181          G .                                                .   240
    AGCAGCTAGAAGATGGACGTACTTTGTCTGACTACAATATTCAAAAGGAGTCTACTCTTC
      Q  L  E  D  G  R  T  L  S  D  Y  N  I  Q  K  E  S  T  L  H

241                         ↓                                   .   300
    ATCTTGTGTTGAGACTTCGTGGTGGTGCTAAGAAAAGGAAGAAGAAGTCTTACACCACTC
      L  V  L  R  L  R  G  G [A  K  K  R  K  K  K  S  Y  T  T  P]

301                                                             .   360
    CCAAGAAGAATAAGCACAAGAGAAAGAAGGTTAAGCTGGCTGTCCTGAAATATTATAAGG
      K  K  N  K  H  K  R  K  K  V  K  L  A  V  L  K  Y  Y  K  V

361                                                             .   420
    TGGATGAGAATGGCAAAATTAGTCGCCTTCGTCGAGAGTGCCCTTCTGATGAATGTGGTG.
      D  E  N  G  K  I  S  R  L  R  R  E  C  P  S  D  E  C  G  A

421                                                             .   480
    CTGGGGTGTTTATGGCAAGTCACTTTGACAGACATTATTGTGGCAAATGTTGTCTGACTT
      G  V  F  M  A  S  H  F  D  R  H  Y  C  G  K  C  C  L  T  Y

481                                                 . TA    ----    540
    ACTGTTTCAACAAACCAGAAGACAAGTAACTGTATGAGTTAATAAAAGACAT-GAACTAAC(A)n
     C  F  N  K  P  E  D  K  *]
```

**Figure 1** cDNA sequence of HUBCEP80. **a**, cDNA sequences isolated from C328 (carcinoma) and F455 (fibroadenoma) cDNA libraries compared with the complete coding sequences of HUBCEP80. Open box: 5′ and 3′ non-translated sequences; hatched box: ubiquitin encoding sequences; solid box: CEP80 encoding sequences. **b**, Combined cDNA sequence is shown with deviations in the published sequence (Lund *et al.*, 1985) shown above and amino acid sequence shown below. Boxed amino acids indicate the CEP80 protein sequence. Arrow indicates a *Dde*I restriction site. The sequence 3′ of this from C328-8 were used as the CEP80-specific probe.

Since neither C328-8 nor the published sequence corresponds to a full length coding sequence, a breast fibroadenoma cDNA library (Sharp *et al.*, manuscript submitted) was screened to isolate full length or overlapping clones which contain the complete sequence. Two cDNAs were isolated which hybridised to both ubiquitin and CEP80 DNA sequences from C328-8. The three overlapping clones together contain the complete coding sequences of HUBCEP80 (Figure 1a). These two latter clones are in agreement with the published sequences at position 188 but differ in the coding region by a silent A→G at position 53 (Figure 1b).

*Expression of EF-1α*

Northern analysis using C328-5 insert DNA as a probe showed that the EF-1α cDNA hybridised to a single RNA species of 1700nt (data not shown), in agreement with published data (Uetsuki *et al.*, 1989). In order to confirm that EF-1α is expressed at higher levels in fibroadenomas than in carcinomas the same probe was hybridised to slot blots containing total RNA from 16 fibroadenomas and 16 carcinomas. The slots were controlled for loading by hybridisation to a probe for total mRNA from cell line MDA-MB-468. An empirically-derived transformation of $Y_D = Y^{0.7692}$ (where $Y_D$ = value measured by laser scanning densitometry) converted the lag and linear phases of a calibration graph, from a direct plot of RNA loaded in μg against $Y_D$, into a straight line through the origin. A similar transformation ($Y_D = Y^{0.7440}$) gave a straight line through the origin for the EF-1α probe hybridised to the control serial dilutions of cell line RNA. Hence, corrected values for EF-1α expression in the tumours could be calculated by a direct ratio (Swillens *et al.*, 1989). A histogram of these results is shown in Figure 2. The mRNA expression of EF-1α is significantly greater in the fibroadenomas than in the carcinomas ($P < 0.01$, Mann-Whitney U test; $H_0$: $\mu_1 = \mu_2$; $H_a$: $\mu_1 \neq \mu_2$). The carcinomas were a non-homogeneous group, which varied histologically from well to poorly differentiated, from $\leqslant 2$ cm in size to $> 5$ cm with skin tethering, and including infiltrating ductal, infiltrating lobular, mucinous and atypical medullary tumours (Table I). Likewise, the fibroadenomas were non-homogenous with respect to the ratio of peri- to intra-canilicular tissue within the tumour. Hence the underlying

**Figure 2** Relative levels of expression of EF-1α in breast carcinomas and fibroadenomas, assayed by RNA slot blot analysis. Breast tumour RNA was prepared and hybridised as described in Materials and methods, and the filters probed with the EF-1α cDNA (C328-5). Laser scanning densitometry data are displayed as a ratio of the signal obtained with the cDNA to the signal obtained with the reverse transcribed total cDNA probe. These data have been adjusted for the response curve of the autoradiographic film, as described in methods. The expression levels in carcinomas and fibroadenomas are significantly different (($P <$ 0.01) Mann-Whitney U test; $H_0$: $\mu_1 = \mu_2$; $H_a$: $\mu_1 \neq \mu_2$) F: fibroadenoma (solid bars); C: carcinoma (open bars).

**Table I** Clinical parameters of carcinomas

| *Sample no.* | *Class* | *Grade* | *Stage* |
|---|---|---|---|
| C10 | T | I | 1 |
| C298 | ID | II | 3 |
| C300 | ID | III | NK |
| C301 | ID | III | 2 |
| C307 | ID | II | 2 |
| C312 | ID | III | 3 |
| C317 | ID | III | 3 |
| C328 | ID | III | 1 |
| C336 | ID | III | 2 |
| C378 | M | II | 3 |
| C410 | ID | III | 2 |
| C446 | ID | III | 3 |
| C670 | AM | III | NK |
| C712 | ID | III | NK |
| C714 | ID | III | 1 |
| C719 | ID | I | 1 |
| C718 | IL | II | 1 |

AM: atypical medullary; ID: infiltrating ductal; IL: infiltrating lobular; M: mucinous; T: tubular; NK: not known.

distribution of gene expression in these tumour types is unknown and cannot be assumed to be normal. Therefore the non-parametric Mann-Whitney U test was used.

Representative *in situ* hybridisation of riboprobes to sections of fixed tissue from carcinomas and fibroadenomas is shown in Figure 3. In both carcinoma and fibroadenoma samples the antisense riboprobe hybridised at a low level to most stromal cells, with higher levels of labelling over normal breast epithelia and blood vessel endothelium. Labelling of the ductular components of the fibroadenomas was relatively even (Figures 3a and 3b), and involved both epithelium and myoepithelium. Stromal cells generally gave a lower level of labelling although this was variable (Figure 3b). The carcinomas showed a greater variation in the labelling of the tumour cells. Areas within ductal carcinoma *in situ* had greater labelling of cells than adjacent invasive tumour (Figure 3c). In both tissue types the sense riboprobe showed only non-specific hybridisation at background levels (Figure 3d).

**Figure 3** **a**, Low power view of a fibroadenoma with a predominantly pericanilicular pattern showing labelling of all ductal components. **b**, Higher power view of an intracanilicular-type duct, with labelling of epithelium and myoepithelium. The stromal cells have a variable labelling pattern. **c**, Carcinoma 312 with an area of ductal carcinoma *in situ* showing prominent labelling of cells. Adjacent to it there are small groups of infiltrating tumour cells (arrowed) which show a lower level of labelling. **d**, Fibroadenoma probed with sense riboprobe, there being no specific hybridisation.

*Expression of HUBCEP80*

Northern analysis of poly(A)$^+$RNA from three cell lines (MDA MB 231, MCF 7 & HS578T) showed that a ubiquitin-sequence specific probe detected mRNA species at 2,300, 1,100 and 660nt, whilst the corresponding CEP80 sequence-specific probe detected only the 660nt species (data not shown). These results agree with those obtained by Wiborg *et al.* (1985). The 660nt species was by far the most abundant of the three species in these cell lines. Northern analysis of total RNA from seven fibroadenomas and seven carcinomas showed that CEP80 sequences are expressed significantly more in fibroadenomas than in carcinomas (Mann-Whitney U test ($P<0.02$) $H_0$: $\mu_1=\mu_2$; $H_a$: $\mu_1 \neq \mu_2$) (Figure 4).

### Discussion

Differential screening of a carcinoma cDNA library with probes derived from ten different, arbitrary fibroadenoma/carcinoma pairings identified 16 clones that were consistently expressed to different levels in one tumour type compared with the other. Of these, two were found to encode sequences for the translation-associated proteins EF-1α and CEP80. Both these sequences had higher levels of expression in benign (fibroadenoma) than in malignant (carcinoma) tumours, as seen in the RNA slot blot analysis for EF-1α (Figure 2) and Northern analysis for CEP80 (Figure 4). In addition, we have previously found enhanced expression of another translation-associated protein, P2, in benign compared with malignant tumours (Sharp *et al.*, 1990). *In situ* hybridisation of EF-1α riboprobes to carcinoma and fibroadenoma tissues showed that the higher levels of expression resided mainly in the tumour cells or normal glandular structures rather than in the stromal cells, although the fibroadenomas did contain varying numbers of stromal cells with higher levels of expression. The opposite was the case for stromelysin-3, a new member of the family of metalloproteinases which degrade the extracellular matrix, where the gene was specifically expressed in the stromal cells surrounding invasive breast carcinomas (Basset *et al.*, 1990).

Since quantification of *in situ* hybridisation is difficult it was not possible to determine the relative levels of expression of EF-1α mRNAs in carcinomas and fibroadenomas by this method. However, a number of interesting features could be observed. The frankly invasive carcinoma cells of C312 showed lower expression than cells of adjacent ductal carcinoma *in situ* which is of interest since the intraductal carcinoma represents an earlier stage of the disease.

There is one report of a novel sequence, pGM21, isolated by differential screening of a subtractive cDNA library constructed from a poorly metastatic rat mammary adenocarcinoma cell line (DMBA8) and a highly metastatic variant line (DMBA8 ascites) and found to be associated with high metastatic potential (Phillips *et al.*, 1990). This gene was said to contain a 45 nucleotide segment homologous to human EF-1α cDNA (Brands *et al.*, 1986), however, this is incorrect and there is no significant homology of pGM21 to any published sequence. A retraction to this effect will be published (I.A. Ramshaw, personal communication).

The process of peptide elongation is fundamental to the function of all living cells and is highly conserved between prokaryotes and eukaryotes. Hence, regulation of the component parts is critical to normal development of the cell. Of the two translation-associated proteins considered here, EF-1α is responsible for targetting the incoming aminoacyl-tRNA to the ribosome which leads to incorporation of the amino acid and elongation of the nascent peptide. Besides its essential role in peptide elongation, EF-1α may also play a key part in the regulation of a number of important cellular functions. For example, EF-1α is found in association with membranes and tubulin (Janssen & Möller, 1988), endoplasmic reticulum (Hayashi *et al.*, 1989), the mitotic apparatus (Ohta *et al.*, 1990) and the cytoskeletal apparatus (Yang *et al.*, 1990). The amount of EF-1α within the cell is controlled

**Figure 4** Relative levels of expression of HUBCEP80 gene in breast carcinomas and fibroadenomas, as shown by Northern blotting. Breast tumour RNA was electrophoresed and blotted according to Materials and methods, and the filters probed with **a**, CEP80 (see Figure 1), stripped and reprobed with **b**, radiolabelled cDNA synthesised by reverse transcription of 4.65 μg oligo(dT) primed total RNA from cell lines MDA MB 231, HS578T, HeLa, fibroadenomas F524, F672 and carcinoma C690 in a ratio of 15: 8: 1: 2.5: 10: 10 as a control for mRNA loading. Sizes of transcripts are in nucleotides. Filters were washed to a stringency of 0.5 × SSC at 65°C. Laser scanning densitometry data, corrected for mRNA loading, are displayed **c**, as a percentage of the highest signal obtained from any tumour – i.e. fibroadenoma F524. The expression levels in carcinomas and fibroadenomas are significantly different ($P<0.02$, Mann-Whitney U test; $H_0$: $\mu_1=\mu_2$; $H_a$: $\mu_1 \neq \mu_2$) F: fibroadenoma (solid bars); C: carcinoma (open bars).

at the transcriptional level as well as translationally and post-translationally.

There is evidence that EF-1α also plays an important role in ageing. During successive passages of mortal fibroblasts there is a decline in the amount of activity of EF-1α as the cells reach the limit of their *in vitro* lifespan (Cavallius *et al.*, 1986). Shepherd *et al.* (1989) reported that *Drosophila melanogaster* have increased longevity when over-expressing an extra copy of EF-1α introduced by P-element plasmid transformation. Our findings of higher levels of EF-1α gene expression in fibroadenomas compared with carcinomas could be related to a greater cell longevity in fibroadenomas, which is observed as an overall greater growth rate in many fibroadenomas (Meyer, 1977).

The second ribosomal protein isolated in this study was the linear ubiquitin adduct HUBCEP80 co-translated as a single polypeptide from a transcript of a natural fusion gene, in which the codon for the terminal glycine of ubiquitin is followed immediately by the initial alanine codon for the 80 amino acid extension protein (CEP80). Northern analysis (Figure 4) showed that the CEP80 sequences were differentially expressed in a similar fashion to EF-1α in the two tumour types (i.e. C328-8 showed higher expression in fibroadenomas than in carcinomas).

CEP80 has been identified as the human homologue of the rat basic ribosomal protein S27a (Redman & Rechsteiner, 1989). The S27a protein is one of about 30 ribosomal proteins found in the small (40S) subunit of eukaryotic ribosomes. The homologue in *Saccharomyces cerevisiae* (CEP76, Özkaynak *et al.*, 1987) is involved in pre-rRNA processing at the step where 20S rRNA is cleaved to 18S rRNA. Mutants with deletions in the *UB13* gene encoding CEP76 in *S. cerevisiae* are deficient in 18S rRNA because the 20S rRNA is degraded instead of being processed (Finley *et al.*, 1989). Induced expression of human HUBCEP80 causes growth inhibition in *S. cerevisiae* (Monia *et al.*, 1989). The CEP80 sequences and not the ubiquitin sequences are responsible for this inhibition. Equally, under-expression of CEPs affects growth, as seen in the slower growth rates of deletion mutants of *UB11*, *UB12* and *UB13* of *S. cerevisiae* (Finley *et al.*, 1989). All these observations suggest that the regulation of CEP production in cells must be critical for normal growth (Monia *et al.*, 1989). The differing levels of CEP80 expression in fibroadenomas and carcinomas possibly reflect aberrant regulation of CEP80 sequences.

In the present study we have identified two translation-associated proteins as being involved in differential mRNA expression in benign and malignant tumours. We have previously described a similar expression profile for a sequence encoding the large ribosomal subunit acidic phosphoprotein P2 and we discussed that this was unlikely to be due to the rates of proliferation of the tumour types compared (Sharp *et al.*, 1990). We have, therefore, presented evidence that a number of key components in the translation machinery show differential expression in malignant breast disease.

We are grateful to the surgeons of the Leicestershire District Health Authority for their co-operation, and to ICI for their financial support. We also thank Dr Kerry Chester for valuable discussion, Dr Paul Senior and Simon Byrne for invaluable help with *in situ* hybridisation technology and Angie Phanopoulos for technical assistance.

### References

ANN, D.K., WU, M.M.J., HUANG, T., CARLSON, D.M. & WU, R. (1988). Retinol-regulated gene expression in human tracheobronchial epithelial cells. *J. Biol. Chem.*, **263**, 3546.

BASSET, P., BELLOCQ, J.P., WOLF, C. & 7 others (1990). A novel metalloproteinase gene specifically expressed in stromal cells of breast carcinomas. *Nature*, **348**, 699.

BLAMEY, R.W., DAVIES, C.J., ELSTON, C.W., JOHNSON, J., HAYBITTLE, J.L. & MAYNARD, P.V. (1979). Prognostic factors in breast cancer: the formation of a prognostic index. *Clin. Oncol.*, **5**, 227.

BRANDS, J.H.G.M., MAASSEN, J.A., VAN HEMERT, F.J., AMONS, R. & MÖLLER, W. (1986). The primary structure of the α subunit of human elongation factor 1. *Eur. J. Biochem.*, **155**, 167.

BRINKLEY, D. & HAYBITTLE, J.L. (1975). The curability of breast cancer. *Lancet*, **ii**, 95.

CAVALLIUS, J., RATTAN, S.I.S. & CLARK, B.F.C. (1986). Changes in activity and amount of active elongation factor 1α in ageing and immortal human fibroblast cultures. *Exp. Gerontol.*, **21**, 149.

CHADWICK, D.E. & LAGARDE, A.E. (1988). Coincidental acquisition of growth autonomy and metastatic potential during the malignant transformation of factor-dependent CCL39 lung fibroblasts. *J. Natl Cancer Inst.*, **80**, 318.

COX, K.H., DELEON, D.H., ANGERER, L.M. & ANGERER, R.C. (1984). Detection of mRNAs in sea urchin embryos by *in situ* hybridization using asymmetric RNA probes. *Dev. Biol.*, **101**, 485.

DEAR, T.N. (1990). Note re: T. Neil Dear *et al.*, Transcriptional down-regulation of a rat gene, WDNM2, in metastatic DMBA-8 cells (*Cancer Res.*, **49**, 5323, (1989)). *Cancer Res.*, **50**, 1667.

DEAR, T.N., RAMSHAW, I.A. & KEFFORD, R.F. (1988). Differential expression of a novel gene, WDNM1, in nonmetastatic rat mammary adenocarcinoma cells. *Cancer Res.*, **48**, 5203.

DEAR, T.N., MCDONALD, D.A. & KEFFORD, R.F. (1989). Transcriptional down-regulation of a rat gene, WDNM2, in metastatic DMBA-8 cells. *Cancer Res.*, **49**, 5323.

ELVIN, P., KERR, I.B., MCARDLE, C.S. & BIRNIE, G.D. (1988). Isolation and preliminary characterisation of cDNA clones representing mRNAs associated with tumour progression and metastasis in colorecal cancer. *Br. J. Cancer*, **57**, 36.

FEINBERG, A.P. & VOGELSTEIN, B. (1983). A technique for radiolabelling DNA restriction endonuclease fragments to high specific activity. *Anal. Biochem.*, **132**, 6.

FELDMAN, M. & EISENBACH, L. (1988). Genes controlling the metastatic phenotype. *Cancer Surv.*, **7**, 555.

FINLEY, D., BARTEL, B. & VARSHAVSKY, A. (1989). The tails of ubiquitin precursors are ribosomal proteins whose fusion to ubiquitin facilitates ribosome biogenesis. *Nature*, **338**, 394.

FISHER, E.R., SASS, R. & FISHER, B. (1984). Pathologic findings from the National Surgical Adjuvant Project for breast cancer (protocol no. 4). Discrimination for tenth year treatment failure. *Cancer*, **53**, 712.

GRUNSTEIN, M. & HOGNESS, D.S. (1975). Colony hybridization: a method for the isolation of cloned DNAs that contain a specific gene. *Proc. Natl Acad. Sci. USA*, **72**, 3961.

HAMADA, J., TAKEICHI, N. & KOBAYASHI, H. (1988). Metastatic capacity and intercellular communication between normal cells and metastatic cell clones derived from a rat mammary adenocarcinoma. *Cancer Res.*, **48**, 5129.

HAYASHI, Y., URADE, R., UTSUMI, S. & KITO, M. (1989). Anchoring of peptide elongation factor EF-1α by phosphatidylinositol at the endoplasmic reticulum membrane. *J. Biochem.*, **106**, 560.

HENNESSY, C., HENRY, J.A., MAY, F.E.B., WESTLEY, B.R., ANGUS, B. & LENNARD, T.W.J. (1991). Expression of the antimetastatic gene nm23 in human breast cancer: an association with good prognosis. *J. Natl Can. Inst.*, **83**, 281.

HERMANEK, P. & SOBIN, L.H. (1987). (eds) TNM *Classification of Malignant Tumours*, 4th edition. Springer-Verlag: Berlin.

JANSSEN, G.M.C. & MÖLLER, W. (1988). Elongation factor 1βγ from *Artemia*. Purification and properties of its subunits. *Eur. J. Biochem.*, **171**, 119.

KRAFT, R., TARDIFF, J., KAUTER, K.S. & LEINWAND, L.A. (1988). Using mini-prep plasmid DNA for sequencing double stranded templates with Sequenase. *BioTechniques*, **6**, 544.

LUND, P.K., MOATS-STAATS, B.M., SIMMONS, J.G. & 4 others (1985). Nucleotide sequence analysis of a cDNA encoding human ubiquitin reveals that ubiquitin is synthesized as a precursor. *J. Biol. Chem.*, **260**, 7609.

MATRISIAN, L.M. & BOWDEN, G.T. (1990). Stromelysin/transin and tumor progression. *Cancer Biol.*, **1**, 107.

MEYER, J.S. (1977). Cell proliferation in normal human breast ducts, fibroadenomas, and other ductal hyperplasia measured by nuclear labeling with tritiated thymidine. *Hum. Pathol.*, **8**, 67.

MONIA, B.P., ECKERS, D.J., JONNALAGADDA, S. & 4 others (1989). Gene synthesis, expression and processing of human ubiquitin carboxyl extension proteins. *J. Biol. Chem.*, **264**, 4093.

MULLINS, D.E. & ROHRLICH, S.T. (1983). The role of proteinases in cellular invasiveness. *Biochim. Biophys. Acta*, **695**, 177.

NICHOLSON, G.L., DULSKI, K.M. & TROSKO, J.E. (1988). Loss of intercellular junctional communication correlates with metastatic potential in mammary adenocarcinoma cells. *Proc. Natl Acad. Sci. USA*, **85**, 473.

OHTA, K., TORIYAMA, M., MIYAZAKI, M. & 4 others (1990). The mitotic apparatus-associated 51-kDa protein from sea urchin eggs is a GTP-binding protein and is immunologically related to yeast polypeptide elongation factor 1α. *J. Biol. Chem.*, **265**, 3240.

ÖZKAYNAK, E., FINLEY, D., SOLOMON, M.J. & VARSHAVSKY, A. (1987). The yeast ubiquitin genes: a family of natural gene fusions. *EMBO. J.*, **6**, 1429.

PHILLIPS, S.M., BENDALL, A.J. & RAMSHAW, I.A. (1990). Isolation of gene associated with high metastatic potential in rat mammary adenocarcinomas. *J. Natl Cancer Inst.*, **82**, 199.

REDMAN, K.L. & RECHSTEINER, M. (1989). Identification of the long ubiquitin extension as ribosomal protein S27a. *Nature*, **338**, 438.

RETZEL, E.F., COLLETT, M.S. & FARAS, A.J. (1980). Enzymatic synthesis of deoxyribonucleic acid by the avian retrovirus reverse transcriptase *in vitro*: optium conditions required for transcription of large ribonucleic acid templates. *Biochemistry*, **19**, 513.

SANGER, F., NICKLEN, S. & COULSON, A.R. (1977). DNA sequencing with chain terminating inhibitors. *Proc. Natl Acad. Sci. USA*, **74**, 5463.

SANGER, F. & COULSON, A.R. (1978). The use of thin acrylamide gels for DNA sequencing. *FEBS Lett.*, **87**, 107.

SENIOR, P.V., CRITCHLEY, D.R., BECK, F., WALKER, R.A. & VARLEY, J.M. (1988). The localisation of laminin mRNA and protein in the postimplantation embryo and placenta of the mouse. An *in situ* hybridization and immunocytochemical study. *Development*, **104**, 431.

SHARP, M.G.F., ADAMS, S.M., ELVIN, P., WALKER, R.A., BRAMMAR, W.J. & VARLEY, J.M. (1990). A sequence previously identified as metastasis-related encodes an acidic ribosomal phosphoprotein P2. *Br. J. Cancer*, **61**, 83.

SHEPHERD, J.C.W., WALLDORF, U., HUG, P. & GEHRING, W.J. (1989). Fruit flies with additional expression of the elongation factor EF-1α live longer. *Proc. Natl Acad. Sci. USA*, **86**, 7520.

STEEG, P.S., BEVILACQUA, G., KOPPER, L. & 4 others (1988). Evidence for a novel gene associated with low tumor metastatic potential. *J. Natl Cancer Inst.*, **80**, 200.

SWILLENS, S., COCHAUX, P. & LECOCQ, R. (1989). A pitfall in the computer-aided quantitation of autoradiograms. *TIBS*, **14**, 440.

UETSUKI, T., NAITO, A., NAGATA, S. & KAZIRO, Y. (1989). Isolation and characterization of the human chromosomal gene for polypeptide elongation factor-1α. *J. Biol. Chem.*, **264**, 5791.

UPDYKE, T.V. & NICHOLSON, G.L. (1986). Malignant melanoma cell lines selected *in vitro* for increased homotypic adhesion properties have increased experimental metastatic potential. *Clin. Exp. Metastasis*, **4**, 273.

WALKER, R.A., SENIOR, P.V., JONES, J.L., CRITCHLEY, D.R. & VARLEY, J.M. (1989). An immunohistochemical and *in situ* hybridization study of *c-myc* and *c-erb*B2 expression in primary human breast carcinomas. *J. Pathol.*, **158**, 97.

WALLETT, V., MUTZEL, R., TROLL, H. & 4 others (1990). *Dictyostelium* nucleoside diphosphate kinase highly homologous to nm23 and awd proteins involved in mammalian tumour metastasis and *Drosophila* development. *J. Natl Cancer Inst.*, **82**, 1199.

WHITTAKER, J.L., WALKER, R.A. & VARLEY, J.M. (1986). Differential expression of cellular oncogenes in benign and malignant human breast tissue. *Int. J. Cancer*, **38**, 651.

VARLEY, J.M., SWALLOW, J.E., BRAMMAR, W.J., WHITTAKER, J.L. & WALKER, R.A. (1987). Alterations to either c-*erb*B-2 (*neu*) or *c-myc* proto-oncogenes in breast carcinomas correlate with poor short-term prognosis. *Oncogene*, **1**, 423.

WIBORG, O., PEDERSEN, M.S., WIND, A., BERGLUND, L.E., MARCKER, K.A. & VUUST, J. (1985). The human ubiquitin multigene family: some genes contain multiple directly repeated ubiquitin coding sequences. *EMBO J.*, **4**, 755.

YANG, F., DEMMA, M., WARREN, V., DHARNAWARDGABE, S. & CONDEELIS, J. (1990). Identification of an actin-binding protein from *Dictyostelium* as elongation factor 1a. *Nature*, **347**, 494.

ZUCKER, S. (1988). A critical appraisal of the role of proteolytic enzymes in cancer invasion: emphasis on tumor surface proteinases. *Cancer Invest.*, **6**, 219.